# Robustness of plasmon phased array nanoantennas to disorder

**DOI:** 10.1038/srep10911

**Published:** 2015-06-03

**Authors:** Felipe Bernal Arango, Rutger Thijssen, Benjamin Brenny, Toon Coenen, A. Femius Koenderink

**Affiliations:** 1 Center for Nanophotonics, FOM Institute AMOLF, Science Park 104, NL-1098XG Amsterdam, The Netherlands

## Abstract

We present cathodoluminescence experiments that quantify the response of plasmonic Yagi-Uda antennas fabricated on one-dimensional silicon nitride waveguides as function of electron beam excitation position and emission wavelength. At the near-infrared antenna design wavelength cathodoluminescence signal robustly is strongest when exciting the antenna at the reflector element. Yet at just slightly shorter wavelengths the signal is highly variable from antenna to antenna and wavelength to wavelength. Hypothesizing that fabrication randomness is at play, we analyze the resilience of plasmon Yagi-Uda antennas to varations in element size of just 5 nm. While in our calculations the appearance of directivity is robust, both the obtained highest directivity and the wavelength at which it occurs vary markedly between realizations. The calculated local density of states is invariably high at the reflector for the design wavelength, but varies dramatically in spatial distribution for shorter wavelengths, consistent with the cathodoluminescence experiments.

Optical phased array antennas^1^ are nanoscale scattering structures that promise to improve single molecule spectroscopy^2^,^3^, sensing of analytes in attoliter volumes^4^, and to provide ultracompact unidirectional couplers of light into, e.g., waveguide circuits^5^,^6^,^7^. Phased array antennas steer light by interference using two control mechanisms. First, as building blocks one uses subwavelength scatterers with a resonant response, thereby gaining control over the complex amplitude and phase of their scattering^1^. Second, proper geometrical arrangement at subwavelength distances introduces retardation between scattering centers. Thereby interference leads to directionality^8^, even if each single element is not strongly directional. A key example in the field of plasmonics is the so-called Yagi-Uda antenna^9^,^10^,^11^,^12^,^13^. This unidirectional antenna consists of a feed element driven by a local emitter like a single molecule, surrounded by a linear chain of particles that act as “directors”, and a red-detuned element that acts as “reflector” by canceling emission of the feed into one half-space. This type of antenna was demonstrated to indeed yield directed scattering and emission^14^,^15^,^16^ and conversely high signal gain locally at the feed element in receiving mode^17^,^18^. A promising use of phased array antennas is to integrate them with dielectric waveguides^5^,^6^,^7^. This constitutes a route to use nanoplasmonics in integrated optical information processing, or lab-on-chip settings^5^,^6^,^7^,^19^,^20^,^21^.

Robustness to fabrication disorder is a key requirement for nano-optics. Compared to plasmonic antennas that rely on narrow gaps, phased array antennas could be more resilient, since functionality mainly relies on retardation effects that are not sensitive to nanometer displacements. In this paper we present an analysis of the robustness of Yagi-Uda antennas to disorder on basis of two figures of merit, namely, directivity, and “local density of states” (LDOS) - also often referred to as Purcell enhancement^22^. This study includes experiments in which we measured the total radiated power from waveguide-integrated Yagi-Uda antennas that are locally excited using cathodoluminescence spectroscopy (CL). CL excitation maps, that are generally agreed to correspond to LDOS for plasmonic structures, are remarkably strongly dependent on antenna geometry and wavelength. In this work, first we report on these experiments, demonstrating that CL excitation maps are comparatively robust in the infrared (750 nm and above), yet highly variable at wavelengths to the blue of the antenna design frequency. We explain the variability by a disorder analysis. A Monte Carlo study of antenna robustness against 5 nm (5%) particle size fluctuations shows that directivity is robust, but directivity performance varies strongly from realization to realization. Calculated LDOS maps are robust at the infrared design wavelength, but highly variable at shorter wavelengths, consistent with our CL data.

## Experiment

Cathodoluminescence microscopy relies on light generated by fast electrons in a scanning electron microscope. To obtain a compatible sample geometry we choose a silicon rich Si_3_N_4_ membrane (100 nm thick, Norcada Inc.) as substrate. This presents two advantages. First, thin membranes are preferred for their low intrinsic background luminescence and for having sufficient electron transparency to avoid charging, which would decrease the electron beam spatial accuracy. Second, Si_3_N_4_ membranes have the advantage of presenting a modest perturbation of the antenna phased array physics, as opposed to the commonly used Si as CL substrate. The Si_3_N_4_ membranes supports a TE waveguide mode, making our work directly comparable to recent optical studies of waveguide coupled Yagi-Uda antennas on Si_3_N_4_ on quartz^7^.

**Figure 1:**
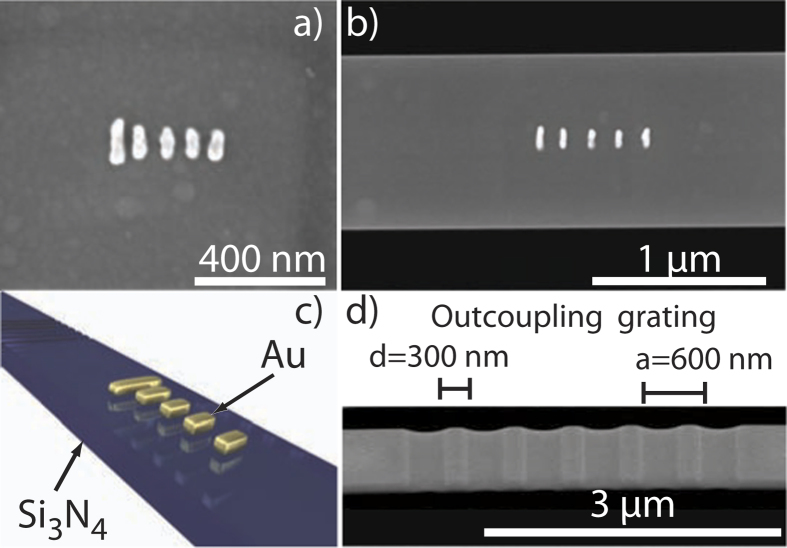
Scanning electron microscope images of **a**) the smallest Yagi-Uda antenna used (YU5) and **b**) a Yagi-Uda antenna on a bridge Si_3_N_4_ waveguide with 1000 nm width. **c**) Sketch of sample geometry. Outcoupling gratings are fabricated in the waveguide using focused ion beam milling at 6 *μ*m distance from the antenna, on each side. The gratings in **d**) have a pitch of 600 nm and a duty cycle of 50%.

The fabrication process has two steps. First, we use electron beam lithography to define gold Yagi-Uda antennas. In brief, ZEP-520 A is spun on the membrane, and we perform electron beam exposure (Raith e-line, 20 keV), physical vapor deposition of gold and lift off (see Fig. 1a). We fabricated Yagi-Uda antennas composed of five elements, a reflector, feed, and 3 directors^14^, at various geometrical parameters. After the electron beam lithography step is completed, the second fabrication step is effectuated by focused ion beam (FIB) milling (FEI Helios) (see Fig. 1b). In this stage we pattern the Si_3_N_4_ around the antennas. We mill 5 *μ*m wide, 20 *μ*m long rectangular holes around the antenna, leaving a 1 *μ*m wide strip, to ensure that the environment that the antennas couple to is a well-defined 1D dielectric waveguide. The waveguide extends 10 *μ*m away from the antenna in both directions. The antennas can couple to the fundamental TE mode of the strip (effective index 1.35 from finite element simulations). According to Ref. 7, the majority of radiation emitted by the Yagi-Uda antenna will funnel into the waveguide. To extract all the light, i.e., also the light coupled to guided modes in the waveguide, outcoupling gratings are defined on both ends, 6 microns away from the antenna (Fig. 1c,d). The grating has a duty cycle of 50%, following design rules for fiber-to-waveguide couplers for minimum back reflection, and high outcoupling^23^. Based on the design analysis by Taillaert *et al.*^23^ we expect efficient outcoupling into the upper half space that our mirror collects with only minor variations in efficiency over the wavelength bandwidth of the experiment (less than 10% efficiency variation expected). We present data for five antennas, with actual antenna dimensions measured after fabrication by scanning electron microscopy, as summarized in Table 1. Antennas 1 to 4 start from a basic design, but with all dimensions scaled by 10% from one to the next. Accordingly, one expects approximately identical CL response, but progressively redshifted. For the fifth antenna, element sizes are comparable to those of antenna 3, but with inter-element spacing reduced by a factor 0.6.

We measured cathodoluminescence using a scanning electron microscope (FEI XL30) in which samples are irradiated by a 30 keV electron beam with a current of ~1 nA and ~5 nm spot size, as reported in^24^,^25^. Luminescence generated by the electron beam is collected by a parabolic mirror that integrates over 1.46*π* sr solid angle, and a large ~25 × 25 *μ*m^2^ collection area encompassing both outcoupling gratings. The cathodoluminescence signal from our sample is dominantly from plasmons induced in the metal nanoparticles by the fast incident electrons^16^,^26^,^27^,^28^,^29^, which subsequently radiate out as light. We spectrally resolve the broad CL signal with a spectrometer and a Princeton Instruments Si CCD detector to obtain wavelength-resolved high resolution spatial maps in the visible frequency spectral range. Count levels on the spectrometer CCD at bright antenna locations were about ~400 analog-digital units per second. As part of the measurement protocol we subtract background signal from unpatterned Si_3_N_4_. The system response function is corrected for by using transition radiation from a single crystalline Au reference substrate^16^,^24^.

### Measurements

**Figure 2:**
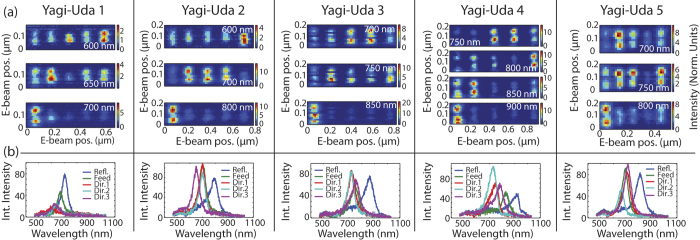
**a**) Cathodoluminescence images of the five Yagi-Uda antennas described in Table 1. In these spectrally resolved excitation maps, the signal was acquired upon local excitation, yet integrating over a large area encompassing antennas and outcoupling gratings, and over 1.46*π* sr solid angle, while the wavelength was selected by a spectrometer. Intensity is relative to the transition radiation from unpatterned single crystalline gold. For each figure, the antenna is oriented with the reflector on the left, followed by feed and three directors. For each antenna, select wavelengths sorted from blue to red are shown as annotated. Panels **b**) show integrated spectra per antenna-element, as obtained from integrating the signal from the individual antenna elements.

Figure 2a shows CL excitation maps measured on five different antennas. When the electron beam is positioned on the antenna elements, we find a strong cathodoluminescence signal. For each plasmon particle, the cathodoluminescence signal is maximum at the extremities. That all the rods light up at both ends but not their center is consistent with the notion that the elements support in-plane electric dipolar resonances. Such in-plane dipole moments can be driven by the out-of-plane incident electrons only when impinging off-center.

Remarkably, the magnitude of the response of different rods in a given antenna is very different, and moreover the distribution of CL intensity varies significantly with wavelength. In general for all antennas we find that the longest element, i.e., the reflector, is most excitable at long wavelengths, while the shorter feed and director elements only become easily excitable at shorter wavelengths. This behavior is reciprocal to the shifting in local intensity distributions versus wavelength for plasmon chains driven by on-axis incident plane waves^7^,^17^. We find that the precise order along the wavelength axis in which directors progressively increase in intensity due to e-beam excitation changes significantly from antenna to antenna. To quantify the spectral response of each individual antenna element we integrate CL signal over areas of 4 × 4 pixels at the rod extremities. Figure 2b shows the spectral dependence of this integrated response. All antenna elements show clear resonances, with full width half maximum around ~60 nm, and resonance frequencies between 600 nm and 900 nm. In all data sets the resonance wavelengths red shift with increasing element size, so that reflectors are distinctly redshifted compared to all other elements. Besides the main resonance, all rods show a small peak around *λ* = 519 nm which we attribute to an out-of-plane dipole resonance.

**Figure 3:**
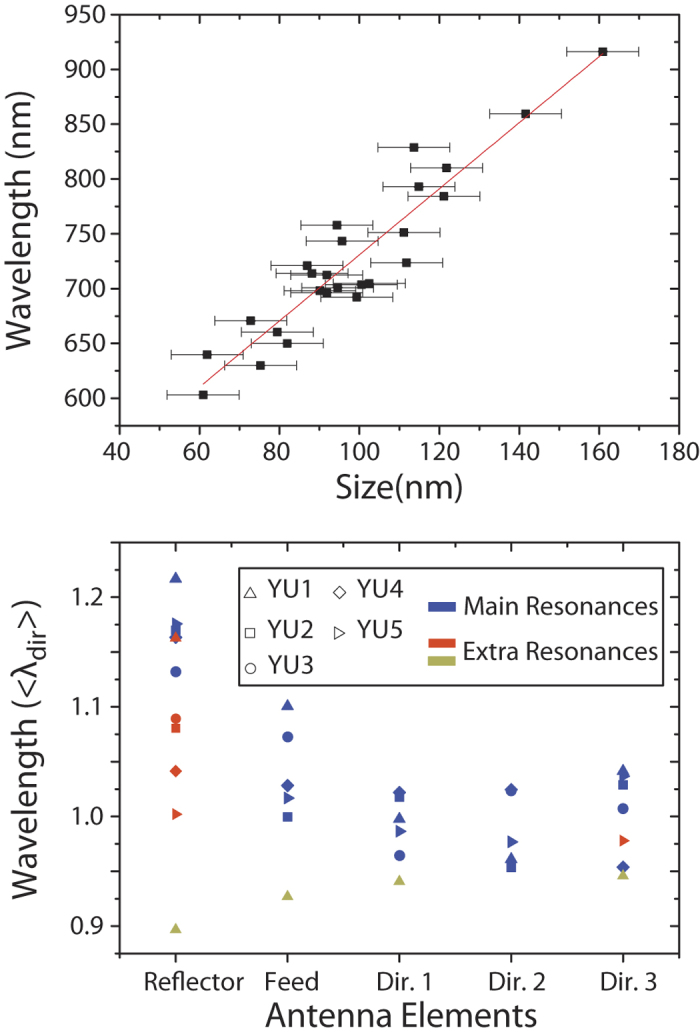
**a**) Wavelength of main resonance peak for every antenna element as a function of its length. **b**) Wavelength of the main and secondary resonances extracted form CL measurements for every element in the different antennas. The wavelengths are normalized to the average main resonance wavelength of the directors in every antenna. Secondary resonances of the reflector (and feed) are close to the director resonance frequency, indicating hybridization.

To first order, the resonance frequency for each element simply varies with rod length as derived for dipole antennas in Ref. 30. Figure 3a shows the extracted main resonance wavelength for all antenna elements as function of element length, obtained using Lorentzian spectral decomposition. The figure shows an approximately linear dependence of resonance wavelength on rod length, consistent with earlier scattering experiments on single rods^18^,^31^,^32^. But, in contrast to those experiments, here the single antenna elements were probed *while* coupled to other antenna elements. Due to the inter-element coupling, an additional shoulder appears in the spectra of those elements that have a resonance located far from the mean resonance wavelength of the antenna. Figure 3b shows both the main and the secondary extracted peak wavelengths for all five elements of all five antennas, sorted by the position of the element in the array. In order to compare all antennas in one plot, the reported wavelengths for each antenna element are normalized to the mean resonance wavelength of just the directors in that antenna. The directors and feed element show usually only one main resonance (blue symbols) and in just few cases a small shoulder (red and green symbols). The reflector element in all cases has its main resonance well redshifted from that of the directors, yet it also presents a clearly blueshifted shoulder. This shoulder, which is much closer to the director element resonance, indicates that the director resonance is driven when exciting the reflector element through dipole-dipole coupling. The hybridization process is easiest to discern in the spectral response of the reflector, since the reflector element is strongly red-shifted compared to all other antenna elements. This strongly red-shifted response also makes the reflector the only excitable element at long wavelengths in all CL maps (Fig. 2a). For shorter wavelengths there are large variations in the spatial maps between antennas over different wavelengths, even though the same behavior is expected *a priori*, modulo a frequency scaling with antenna size. While for single plasmon rods small variations in size are not expected to yield such large differences, we hypothesize that geometrical disorder may induce large variations in LDOS, and hence CL intensity maps, of phased array antennas of coupled elements.

### Monte Carlo study of random disorder

**Figure 4:**
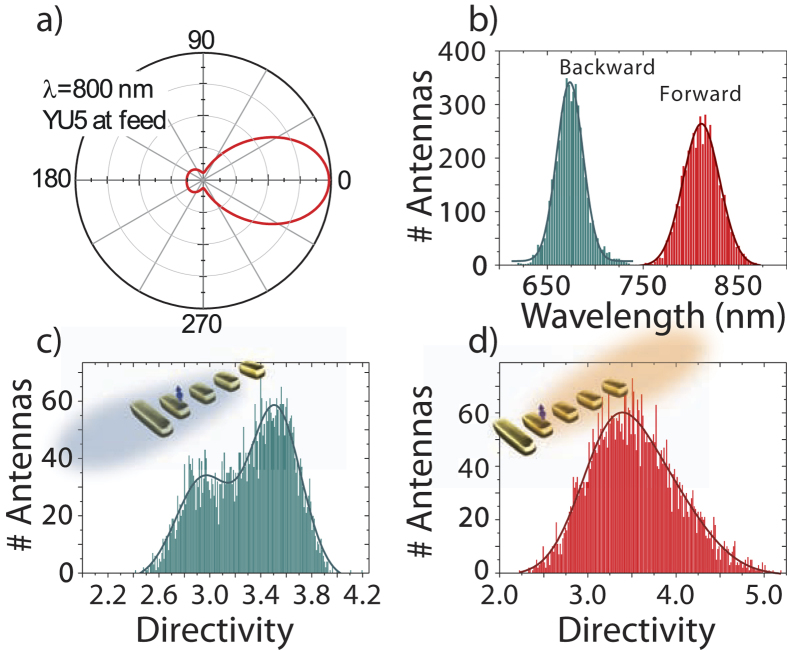
**a**) Polar plot of radiation calculated for ideal YU5 antenna (in free space) driven at 800 nm, with an electric dipole at the feed element (5 nm above the feed, at its edge). The antenna has a directivity of 3.3. (Linear radial scale). Panels (**b–d**): antenna properties for 5000 realizations in which each element of YU5 has a random variation in size (radius measured along major axis) chosen from a normal distribution with 2.5 nm standard deviation. **b**) shows the distribution of wavelengths at which the directivity is maximum in either the forward (red) or backward direction (cyan). **c**) and **d**) show the distributions of maximum directivity for backward and forward radiation, respectively.

To sample the sensitivity of Yagi-Uda antennas to intrinsic fabrication randomness we performed a Monte Carlo study, using as basic geometric design the dimensions of antenna YU5 described in Table 1. We resort to the commonly used electrodynamic point dipole approximation, to rapidly obtain results for many different antennas of randomized geometry around the basic design. The point dipole calculation, as explained in Ref. 7,22, is performed on antennas described by electrically polarizable prolate objects^33^ by using the following self-consistent point dipole expression:


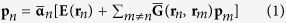


where **p**_*n*_ and ***ᾱ***_*n*_ are the induced dipolar moment and polarizability of the rod element *n*, respectively. An incident field **E**(**r**_*n*_) with frequency *ω* drives the rods located at **r**_*n*_. The Green function **G–**(**r**_*n*_,**r**_*m*_) describes the environment while the randomness is introduced through the polarizability of each element ***ᾱ***_*n*_ which changes in strength and resonance wavelength with the size of the major and minor axes of the prolate spheroid. While the minor axis of the particle is kept constant, the radius of the long axis is changed according to a normal distribution of 2.5 nm standard deviation (just over 5 nm FWHM). Radiation damping and depolarization are included in the polarizability tensors. As driving field **E**(**r**_*n*_) we use localized excitation by a dipolar source (z-oriented, placed 5 nm above the edge of a plasmon rod) thereby mimicking CL excitation^29^. We use directivity and radiative local density of states (LDOS) as figures of merit for antenna performance. Directivity, the figure of merit that Yagi-Uda antennas are designed to improve, is defined as^8^ *D* = max(4*πU* (*θ*,*ϕ*)/*P*_*tot*_), where *U* (*θ*,*ϕ*) is the radiated intensity per steradian and *P*_*tot*_ is the total emitted power. Thus, a perfectly isotropic source has *D* = 1 and a dipolar source which radiates preferentially perpendicular to its axis has *D* = 1.64. The Yagi-Uda antenna design is calculated to have a large forward directivity of *D* = 3.3 at wavelengths around 800 nm (Fig. 4a), with a reversal in the radiation pattern leading to a large backward directivity at 670 nm. The second quantity we will discuss is the radiative LDOS, which is given by the total emitted power at fixed current strength for the localized driving^34^. Since LDOS simulations with and without substrate agreed well for several geometries, we use free space as embedding medium. This increases computation speed and allows us to sample 5000 antenna realizations.

Results for directivity of all antennas when driven at the feed element are shown as histograms in Fig. 4b–d. Panel b) shows the distribution of wavelengths at which maximum forward and backward directivity occurs, while panels c) and d) show histograms of the maximum directivity obtained in either direction. All antenna realizations consistently show their maximum forward emission in a band centered around 810 nm, with a spread of 40 nm in peak wavelength around the design wavelength. Likewise, all antennas realizations show backward beaming, with maximum backward directivity at blue shifted wavelengths, in a 25 nm bandwidth window around 675 nm. Thus the overall behavior of the design that should show strong forward beaming at red wavelengths and reversal to backwards beaming at blueer wavelengths is robust. However, Fig. 4c,d show that attained directivities for both the forward and backward band show a large spread in values from around 2.5 to over 4. In general these results indicate that while obtaining directivity using Yagi Uda antennas is robust in the face of particle size disorder, reasonable control over actual performance (value of directivity) would require tight control over particle geometry.

**Figure 5:**
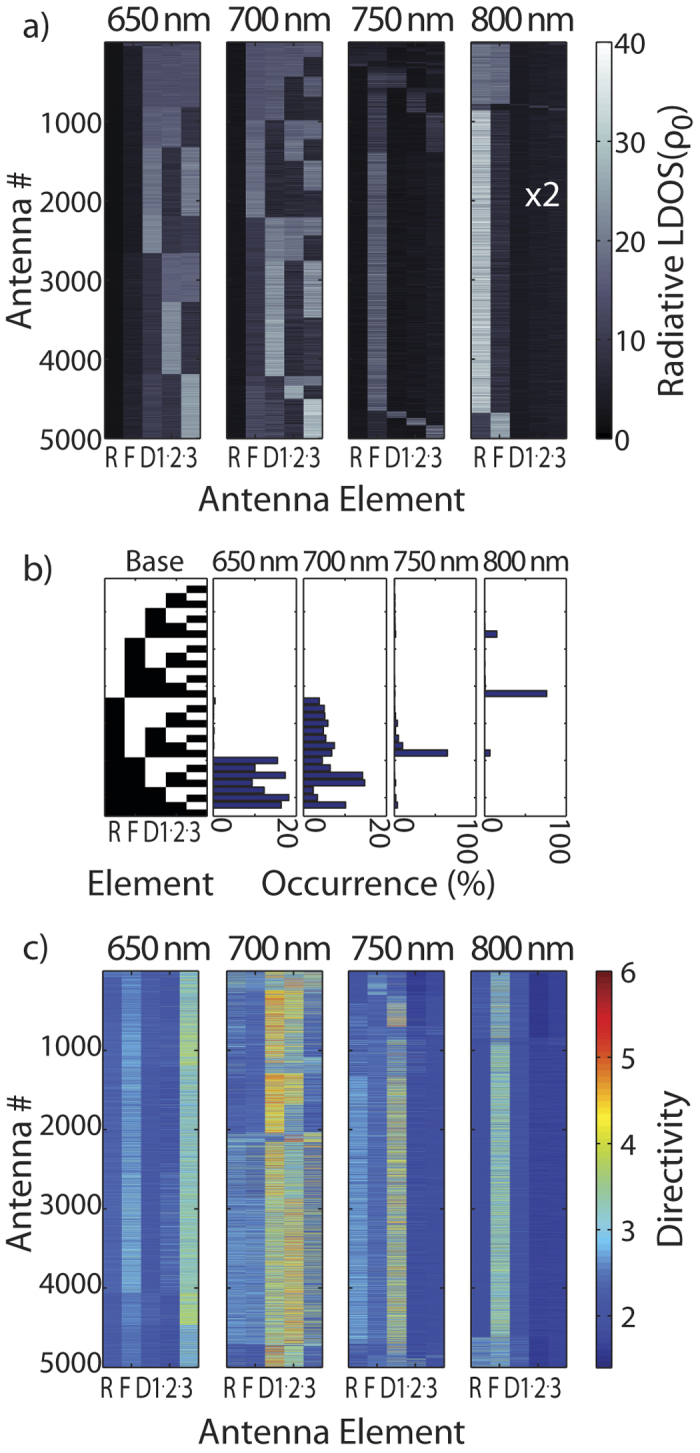
**a**) Radiative LDOS calculated for excitations at every one of the elements of the antennas, for 5000 realizations of antennas with random changes in size of all their elements (labeled as *R,F,D1 2 3* for reflector, feed and directors 1 to 3). **b**) Organizational base used to sort patterns. Histograms with the frequency of occurrence of each pattern as digitized from panels **a**) by thresholding. For the organizational base we depict ‘On” (bright) as white and “Off” as black. **c**) Directivity of the 5000 antenna realizations, calculated for excitation at each of the antenna elements. For 800 nm, this pertains to forward directivity. At shorter wavelenghts, the antennas show backward directivity when driven at the feed.

We now turn to the distribution in radiative LDOS enhancement over the same family of disordered Yagi-Uda antennas. Figure 5 shows the radiative LDOS for driving positions located at a height of 5 nm above the edge of each element of the Yagi-Uda antenna. Changes in the assumed height cause an overall re-scaling of values but no changes in distribution. Each of 5000 realizations is represented by a single 5 pixel horizontal line. Given the robustness of directivity, it is remarkable how sensitive LDOS (and thereby CL maps as those shown in Fig. 2) is to size variations. The different panels in Fig. 5a show radiative LDOS at wavelengths of 650, 700, 750 and 800 nm. In order to improve readability we organize the different antenna realizations according to a binary digitization scheme. This digitization is inspired by Fig. 2, from which it appears that a particle will either be responsive and “bright” in a CL map, or “dark”. As an arbitrary thresholding we label any particle that has radiative LDOS within 20% of that of the highest LDOS particle in the array as bright, and all others as dark. With this definition we can sort antenna realizations using a binary basis as shown in Fig. 5b. This basis is defined as the enumeration of all different possibilities of ‘on’ and ‘off’ states in a 5 element binary array. The sorting allows to assess visually what excitability maps should be predominant.

Figure 5a shows the calculated radiative LDOS as grayscale on all 5000 antennas, sorted according to the digitization base in Fig. 5b. In order to assess how many antennas would be quantified to correspond to a certain five digit binary “on-off” pattern Fig. 5b shows histograms with the frequency of occurrence of each pattern. At the lowest frequency (800 nm wavelength) we find that in above 90% of realizations, the reflector is the dominant excitable element. A few realizations where both reflector and feed, or just the feed element are highly excitable are also found. For shorter wavelengths, the distribution of excitation patterns is far more diverse. Consistent features are that below 800 nm wavelength the reflector is not excitable, and that the LDOS increasingly shifts from feed element (the dominant element in 70% of the realizations at 750 nm) to the directors as 650 nm is approached. In the 650 to 700 nm spectral range, the excitability strongly differs from antenna to antenna realization and is spread over the entire set of directors. Broadly speaking, these simulations are consistent with our experimental observations that at red wavelengths CL intensity always peaks on the reflector end of the antenna, and that at shorter wavelengths the measured CL intensity maps show patterns spread out over all directors with large differences between antennas.

A final question we examine is if LDOS maps have predictive power for directivity, which would imply that CL maps quantify antenna beaming. In Fig. 5c we present directivity for driving at each antenna element for the same antennas as in Fig. 5a, ordered in the same sequence. Thereby a direct side-by-side comparison is possible. In the design band, i.e., at 800 nm all antennas show forward directivity when driven at the feed (as designed), except for those realizations in which the reflector shows no clear LDOS signature. When going to the shorter wavelengths, the feed element consistently provides no directivity, until one reaches the band around 650 nm at which point the antenna is backward directive. This result indicates the robustness of the flip in directivity with increasing frequency that is also evident in Fig. 4. At intermediate wavelengths many realizations fortuitously show high directivity when driving occurs at a director, though with no clear correlation to LDOS. At 700 nm, for instance, directivity is high for excitation at elements 3 and 4 almost irrespective of the antenna realization, although antenna realizations have very different LDOS maps. We conclude that local mapping of LDOS with CL has modest predictive power over the performance as directional antenna in the actual design band, and essentially does not correlate with the (fortuitous) directivity in the shorter wavelength band. Furthermore it should be noted that at the design wavelength, the antenna feed element does give high directivity but is never collocated with highest LDOS, which is instead found on the reflector element. This indicates that the Yagi-Uda design is not an optimal choice for simultaneous control of Purcell enhancement and directivity. This is a generic feature of plasmonic Yagi-Uda antennas, independent of the waveguide geometry. The physics is that optimum directional operation has two requirements. First, one needs operation wavelengths not on, but to the red of, the bare director/feed resonance frequency, in order to excite a transverse particle chain guided mode^13^. At the same time, to make emission unidirectional, the red detuned reflector is chosen such that it provides destructive interference with direct radiation from the feed in one half space. This intrinsically limits total radiated power at given driving dipole strength, or in other words, LDOS. We refer the reader to Ref. 13 for a deeper analysis of the intrinsically poor LDOS enhancement at the feed element in the case of Yagi-Uda antennas in free space. This limitation may be overcome by replacing the feed with a gap antenna.

## Conclusions

We reported cathodoluminescence spectroscopy measurements of phased array antennas fabricated on 1D Si_3_N_4_ waveguides. The data clearly show in-plane electric dipole resonances in the individual elements. To first order the resonance wavelengths per element simply follow the expected size relation for single elements. However, spectra per element also show features of hybridization. We observe large variations in CL signal strength at wavelengths shorter than 750 nm. We performed a Monte Carlo study of radiative LDOS and directivity for antennas modeled as point dipoles with randomly varying particle size. We find that directivity features are fairly robust at the design wavelength. Especially at shorter wavelengths, both directivity and LDOS vary highly from antenna realization to antenna realization. This study highlights the importance of studying robustness to disorder in plasmonic designs of phased array antennas, especially since different figures of merits will be very differently affected.

## Additional Information

**How to cite this article**: Arango, F. B. *et al.* Robustness of plasmon phased array nanoantennas to disorder. *Sci. Rep.* **5**, 10911; doi: 10.1038/srep10911 (2015).

## Figures and Tables

**Table 1:** Measured dimensions (electron microscopy, ±5 nm) of the five Yagi-Uda antennas on which we report CLdata.

**Size [nm]**	**YU1**	**YU2**	**YU3**	**YU4**	**YU5**
Reflector	93	125	130	150	126
Feed	70	100	97	111	105
Director	60	86	84	100	95
Spacing [nm]	YU1	YU2	YU3	YU4	YU5
Reflector to feed	119	130	158	170	89
Feed to director	140	160	183	208	100
Director pitch	137	160	183	208	99
